# Small-Molecule Analysis Based on DNA Strand Displacement Using a Bacteriorhodopsin Photoelectric Transducer: Taking ATP as an Example

**DOI:** 10.3390/s23177453

**Published:** 2023-08-27

**Authors:** Hsiu-Mei Chen, Wen-Chang Wang, Hong-Ren Chen

**Affiliations:** 1 Department of Chemical Engineering, National Taiwan University of Science and Technology, Taipei 10607, Taiwan; 2 The Ph.D. Program for Translational Medicine, College of Medical Science and Technology, Taipei Medical University, Taipei 11031, Taiwan

**Keywords:** ATP, bacteriorhodopsin, photoelectric biosensor, purple membrane, small molecule, strand displacement

## Abstract

A uniformly oriented purple membrane (PM) monolayer containing photoactive bacteriorhodopsin has recently been applied as a sensitive photoelectric transducer to assay color proteins and microbes quantitatively. This study extends its application to detecting small molecules, using adenosine triphosphate (ATP) as an example. A reverse detection method is used, which employs AuNPs labeling and specific DNA strand displacement. A PM monolayer-coated electrode is first covalently conjugated with an ATP-specific nucleic acid aptamer and then hybridized with another gold nanoparticle-labeled nucleic acid strand with a sequence that is partially complementary to the ATP aptamer, in order to significantly minimize the photocurrent that is generated by the PM. The resulting ATP-sensing chip restores its photocurrent production in the presence of ATP, and the photocurrent recovers more effectively as the ATP concentration increases. Direct and single-step ATP detection is achieved in 15 min, with detection limits of 5 nM and a dynamic range of 5 nM–0.1 mM. The sensing chip exhibits high selectivity against other ATP analogs and is satisfactorily stable in storage. The ATP-sensing chip is used to assay bacterial populations and achieves a detection limit for *Bacillus subtilis* and *Escherichia coli* of 10^2^ and 10^3^ CFU/mL, respectively. The demonstration shows that a variety of small molecules can be simultaneously quantified using PM-based biosensors.

## 1. Introduction

Bacteriorhodopsin (BR) is a transmembrane protein that constitutes the purple membrane (PM) of *Halobacterium salinarum*. It captures light via its covalently linked retinal molecule and then pumps a proton uni-directionally from the cytoplasmic (CP) side of the membrane to the extracellular (EC) side of the membrane. Using an electrolyte and an external circuit, the resulting photo-induced proton gradient is readily transformed into a photovoltaic force to power electricity [1,2,3]. BR is an ideal biological photoelectric transducer and is used in a variety of biosensors. BR directly detects changes in pH, anesthetics and ion concentrations, and light and radiation intensities [4,5,6,7,8]. A BR-based bio-affinity sensor was first introduced by Knoblauch et al. to detect maltose using maltose-binding proteins and the principle of Förster Resonance Energy Transfer [9]. The study first sequentially layered maltose-binding proteins and dark quenchers on a hybrid quantum dots–BR electrode and then used the composite electrode to detect the presence of maltose by recording the restoration level of the BR photocurrent due to the displacement of dark quenchers by maltose from maltose-binding proteins.

**Figure 1:**
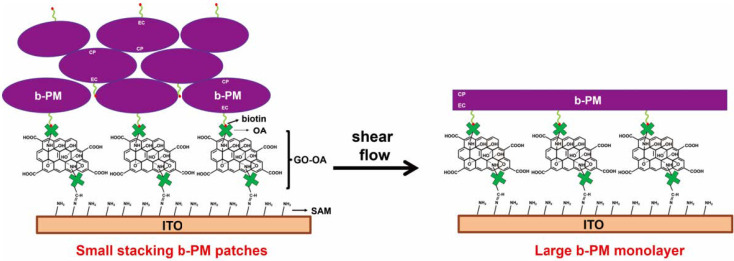
Conceptual illustration of the procedure to fabricate a large b-PM monolayer on ITO using a GO-OA linker and shear flow (figure adapted from [11]).

Recent studies by the authors reported another type of BR-based bio-affinity sensor for the detection of different microorganisms, glycated hemoglobin (HbA1c) and hemoglobin (Hb) using direct, label-free, quantitative, repeatable, sensitive and single-step methods [10,11,12]. The studies used robust, stable and uni-directional BR-linked electrodes as photoelectric transducers. These were fabricated using a combination of a self-assembled monolayer (SAM), covalent linking and avidin–biotin affinity adsorption techniques. In short, the PM was first modified with biotin on its EC side and then affinity-adsorbed on an SAM-aminated electrode using either oxidized avidin (OA) [10] or OA-linked graphene oxide (GO-OA) [11,12] as the linker. As a result, BR was steadily and uni-directionally attached to the electrode to give optimum photoelectric performance and good long-term stability. All BR molecules were coated with the EC side facing the electrode, so the fabrication scheme did not cancel opposite proton fluxes and dipole moments in counter-oriented PM layers and provided the best orientation for BR to generate the greatest photocurrents [13]. Especially, the further addition of double-sided graphene oxide (GO) sheets and shear flow to the scheme yielded a high-coverage and uniformly oriented PM monolayer fabrication on the electrode, as illustrated in Figure 1, facilitating and securing subsequent material attachment [11,12]. Using effective conjugation chemistry, a variety of molecules were covalently linked on top of the PM monolayer, including biotin derivatives, nucleic acid aptamers and *Staphylococcus aureus* protein A. Biotinylated antibodies and unconjugated antibodies were then respectively uni-directionally adsorbed on avidin/NeutrAvidin [10] and protein A [11] and were used to detect different microbes immunologically. All microorganisms were assayed with a limit of detection (LOD) of 1 CFU/10 mL [10,11]. HbA1c and Hb were also identified with their respective specific nucleic acid aptamers covalently conjugated on the PM monolayer, both with an LOD ≤ 0.1 μg/mL. These two aptamer-coated biosensors maintained their sensing and photoelectric activity when stored at 4 °C for 28 days. For clinical samples, the HbA1c levels that weredetermined using these two sensors correlated well with the standard method results [12].

Small molecules are defined as organic compounds with a low molecular weight, which may be natural or artificial. With a great variety of functions or applications, they play essential roles in many diverse fields, such as agriculture, environment, food, healthcare, pharmaceuticals and other industries. The traditional ways to detect small molecules mainly involve chromatography coupled with spectrophotometry or mass spectrometry, which requires expensive equipment, trained personnel and laborious and time-consuming complex sample processing [14]. Immunoassays, especially competitive enzyme-linked immunosorbent assays, have become popular alternatives for small-molecule analysis because they are cost-effective, simple and capable of being performed simultaneously in multiple assays with the advantages of high specificity and sensitivity [14,15]. However, low stability and the high cost of antibodies remain significant issues for immunoassays. On the contrary, specific nucleic acid aptamers, which have high affinity and are generally selected against their respective targets through the systematic evolution of ligands via exponential enrichment, are easily synthesized at a low cost and are relatively stable [16,17,18,19]. In past decades, aptamer-based biosensors, also called aptasensors, have been developed to detect small molecules for a great variety of applications and many different targets [20,21,22,23].

This study aims to extend the application of our previously developed PM monolayer photoelectric transducer to the analysis of small molecules, particularly colorless ones. In the above-mentioned BR-based bio-affinity sensor studies, the targets were all detected presumably based on their light-shielding effects so that their deposition abates the intensity of the incident green light irradiating on the immobilized BR and hence causes the sensor to produce fewer photocurrents. For example, Hb and HbA1c absorb green to yellow light, and microbes scatter visible light. In addition, the binding of those target macro-materials could also restrict the movement of the helices of BR that are responsible for proton pumping, resulting in a decrease in photocurrent generation [10].

**Figure 2:**
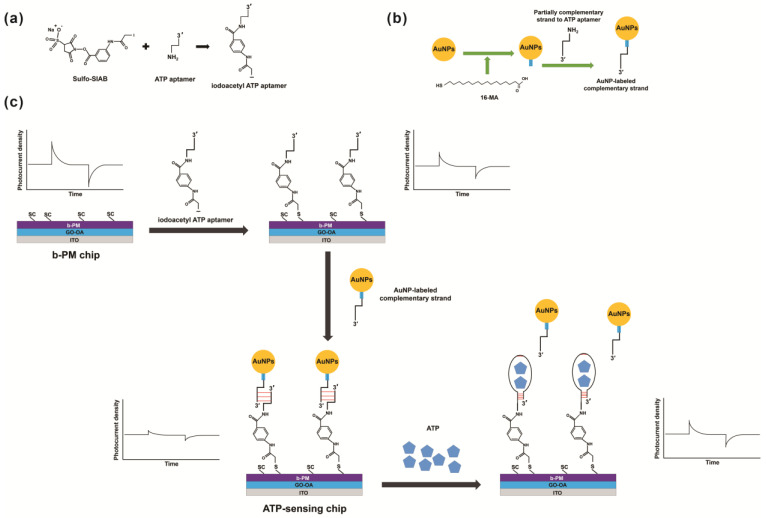
Syntheses of (**a**) iodoacetyl ATP aptamer and (**b**) AuNP-labeled complementary strand. (**c**) Scheme showing the fabrication and ATP detection principle for an ATP-sensing chip.

Small colorless molecules neither absorb nor scatter light, nor do they have a sufficiently high mass density to restrict the movement of BR helices after binding. Hence, it is difficult to detect their presence in samples using the BR-based biosensor sensitively that we previously developed. To overcome this limitation, this study proposes a reverse detection method that employs a DNA strand displacement reaction that occurs when the target binds onto its specific aptamer, and it uses gold nanoparticles (AuNPs) as labels to increase the detection signal. Figure 2 illustrates the proposed detection strategy. The top of the PM monolayer photoelectric transducer is first covalently coated with target-specific aptamers and is then covered with another AuNP-labeled complementary oligonucleotide via DNA hybridization. AuNPs absorb green light [24]. Therefore, their deposition on the PM-coated electrode blocks most light, and photocurrent production is suppressed. If a target is present, it binds with the target-specific aptamers conjugated on the electrode surface. Then, the AuNP-labeled complementary oligonucleotide is released, which restores the capability of the PM monolayer transducer to generate photocurrents. Photocurrent production recovery depends on the target concentration, so quantitative detection is possible.

To demonstrate the feasibility of the proposed detection strategy for small colorless molecules, this study develops a BR-based aptasensor to detect adenosine triphosphate (ATP). ATP is the energy-carrying molecule in all living cells and is involved in many vital metabolic pathways. ATP signals the viability of cells, so its detection is important for bacterial contamination monitoring, bioprocess control and disease diagnosis. Bioluminescence is the most widely used detection principle in current commercial ATP assays because it is highly sensitive and gives fast results. This method uses labile luciferin and luciferase as the reaction substrate and catalyst, respectively, so costly and inconvenient frozen shipments and storage are required. Samples that exhibit background luminescence or contain detergents, ions or other luciferase inhibitors require alternative methods, so the proposed BR-based ATP aptasensor can be a substitute for the bioluminescence assay.

Several other kinds of ATP aptasensors that employ different detection principles have been previously reported, including the AuNP-based colorimetric method [25], the CRISPR-Cas-based platform [26], the electrochemical method [27], molecular beacon-based fluorescence spectroscopy [28], nanocluster-based fluorescence spectroscopy [29], fluorescence resonance energy transfer [30], resonance light scattering [31] and surface-enhanced Raman spectroscopy [32]. This study is the first to quantitatively analyze ATP using BR as the sensor transducer. The preparation and the detection performance of the sensor are reported, as well as the identification of the surface chemicals on the sensing chips.

## 2. Materials and Methods

### 2.1. Materials

b-PM, which is a derivative form of PM with biotin covalently linked on its EC side, and OA were prepared as previously described [33]. 3-Aminopropylphosphonic acid (APPA) was purchased from Acros Organics (Geel, Belgium). Avidin, 1-ethyl-3-(3-dimethylaminopropyl)carbodiimide hydrochloride (EDC), EZ-Link sulfo-NHS-LC-LC-Biotin and sulfosuccinimidyl(4-iodoacetyl)aminobenzoate (Sulfo-SIAB) were purchased from Thermo Fisher Scientific (Waltham, MA, USA). GO powders were purchased from Graphene Supermarket (Ronkonkoma, NY, USA). Indium tin oxide (ITO) glass was purchased from Fang Materials (New Taipei City, Taiwan). DNA oligonucleotides were synthesized by Integrated DNA Technologies (Coralville, IA, USA). ATP, 80 nm AuNPs, 16-mercaptohexadecanoic acid (16-MA) and other chemicals were purchased from Sigma-Aldrich (Burlington, MA, USA). *Escherichia coli* K-12 and *Bacillus subtilis* subsp. *spizizenii* were obtained from the Bioresource Collection and Research Center in Taiwan (Hsinchu City, Taiwan).

### 2.2. Preparation and Chemical Characterization of ATP-Sensing Chip

To produce a b-PM monolayer-coated electrode, which is termed a b-PM chip, an existing protocol was used [11]. An ITO glass electrode was first aminated with 0.1 mM APPA and then sequentially coated with a GO-OA complex linker that was made by mixing GO and OA at a 1:10 weight ratio and with 1.5 mg/mL b-PM. This was washed with laminar shear flow (Reynolds number = 0.9) inside a microfluidic setup.

Prior to the production of the sensing chip, the ATP aptamer (aminoC6-5′-TTT TTT ACC TGG GGG AGT ATT GCG GAG GAA GGT-3′) [34] was modified using excess Sulfo-SIAB and then dialyzed against a 10 mM phosphate buffer (PB) at a pH of 8.5 to yield an iodoacetyl derivative. The partially complementary strand to the ATP aptamer (aminoC6-5′-TTT ACC TTC CTC CGC AAT-3′) was conjugated on AuNPs that were modified with 2.7 mM 16-MA in ethanol for 2 days and then activated using 0.5 mM EDC in a 0.1 M MES buffer at a pH of 5 for 3 h. The processed AuNPs were washed with 10 mM PB at a pH of 7.4 and then suspended in an ATP-binding buffer containing 25 mM Tris-HCl and 300 mM NaCl at a pH of 8.2.

The b-PM chip, which was covered with a monolayer of b-PM, was then coated with the iodoacetyl ATP aptamer at 1 μM, briefly rinsed using the ATP-binding buffer and finally coated with the AuNP-labeled complementary strand at a particle number concentration of 1 pM, followed by another brief rinse with the ATP-binding buffer. The resulting ATP-sensing chip was either subjected to analysis or stored in a 10 mM phosphate buffer containing 150 mM NaCl at a pH of 8.0 at 4 °C.

The surface chemical composition of the sensor chip was determined using a UniNano Tech UniG2D Raman Spectroscope with a 50 mW 532 nm CW laser as the light source.

### 2.3. Detection of ATP and Microbes

An amount of 10 μL of ATP solutions at various concentrations, which were suspended in the ATP-binding buffer, were drop-coated onto the ATP-sensing chip for a direct and single-step assay. After incubation at room temperature for 30 min, the chip was briefly rinsed with the binding buffer, and then the photocurrent was measured immediately.

To assay the amount of ATP in microbes, fresh bacterial cultures were harvested and then suspended at various cell concentrations in the ATP-binding buffer. An amount of 10 μL of 0.4 M HClO_4_ was added to 1 mL of each microbe solution, and this solution was then incubated at 60 °C for 10 min. After centrifugation for 30 min, 10 μL of the supernatant solution was drop-coated onto the ATP-sensing chip for the assay.

To conduct a spike-and-recovery test, field samples of tap water and ground-harvested rainwater from our NTUST campus were tested. First, the calibration curve for each field sample was created by using standards with known concentrations of ATP in the blank field sample. Then, 10 μL of the field sample spiked with ATP at different concentrations was drop-coated onto the ATP-sensing chip and incubated at room temperature for 30 min. The chip was briefly rinsed with its respective blank field sample and then subjected to photocurrent measurement immediately. Finally, the measured ATP concentration in the spiked field sample was determined using its respective calibration curve.

The photocurrent in the b-PM-coated chips was measured inside a cuvette, as described previously [12], using an 80 mW green CW laser as the light source (beam diameter: 3 mm) and 1 mM PB and 10 mM KCl at a pH of 8.5 as the electrolyte. The working b-PM-coated electrode chip was placed against one side wall of the cuvette, with the coating layers facing the light source. A platinum bar was used as the counter electrode and was positioned near the chip. A homemade current amplifier that was connected to a digital oscilloscope was used to measure the photocurrent that was generated by the working electrode chip in real time. Each irradiation cycle comprised 2–3 min of continuous illumination and then 2–3 min of interruptions to illumination. This on-and-off irradiation cycle produced a pair of upward and downward transient photocurrent signals in response to the lights-on and lights-off steps, respectively. The total photocurrent density (I, current/area) for the illuminated chip is defined as the difference between the maximum lights-on and the minimum lights-off signals. The recovery in the photocurrent in the ATP-sensing chip (Equation (1)) is defined as the ratio of the difference between the total photocurrent density in a chip that is coated with ATP ($I_{ATP}$) and in a chip that is coated with the AuNP-labeled complementary strand ($I_{AuNPs}$) to the difference between the total photocurrent density in a chip that is briefly rinsed with the ATP-binding buffer following coating with the ATP aptamer ($I_{buffer}$) and in a chip that is coated with the AuNP-labeled complementary strand ($I_{AuNPs}$).

(1) $$Photocurrent\;recovery\;level\left(\%\right)=\frac{\left(I_{ATP}-I_{AuNPs}\right)}{\left(I_{buffer}-I_{AuNPs}\right)}\times 100\%$$

## 3. Results and Discussion

### 3.1. Chip Fabrication and Characterization

Figure 2 shows the fabrication procedure and ATP detection principle for an ATP-sensing chip. A b-PM monolayer-coated electrode (b-PM chip), which is the photoelectric transducer for the sensor, is the foundation substrate for the sensing chip. It comprises a bottom layer of APPA-aminated ITO glass, a middle double-sided planar GO-OA complex linker layer and the upper uniformly CP-side oriented b-PM monolayer, in which PM is covalently modified with biotin on its EC side and then attached on top of the linker via the avidin–biotin affinity interaction.

To fabricate the monolayer, a post-deposition washing procedure that was previously performed by the authors is used [11]. Microfluidic shear flow over a b-PM drop-coated chip is used to mobilize, segregate, redistribute and reintegrate the original stacked b-PM patches into a large, almost laterally continuous single monolayer. The ATP aptamer is then immobilized onto the pristine b-PM chip surface using a conjugation principle that was previously employed by the authors to attach the non-glycated Hb (HbA0)- and HbA1c-specific aptamers [12]. The 5′-aminated aptamer is first modified into an iodoacetyl derivative with Sulfo-SIAB, which is a heterobifunctional crosslinker containing an iodoacetyl and a sulfosuccinimidyl group at either end. This is then covalently linked onto the exposed CP side of the b-PM chip, which has surface methionine residues [10].

ATP is colorless and too small to cause a sufficiently significant reduction in the photocurrent to allow its quantification after binding onto the b-PM chip, so this study does not use the aptamer-coated b-PM chip to detect ATP directly. A AuNP-labeled strand for which the sequence is partially complementary to the ATP aptamer is bonded onto the aptamer-coated b-PM chip to minimize the photocurrent that is produced by the resulting ATP-sensing chip. The photocurrent is significantly reduced because the 532 nm incident laser light is partially blocked by labeling 80 nm AuNPs on the complementary strand, which the supplier specifies as having a maximum absorbance peak at around 553 nm.

If ATP molecules are present, they competitively bind to the surface ATP aptamers and replace the AuNP-labeled complementary strand. Therefore, the AuNP-labeled complementary strand dissociates, and the photocurrent in the sensing chip is then restored. The extent of strand replacement depends on the amount of ATP, and so does the degree to which the photocurrent is restored. Therefore, the photocurrent recovery level that is calculated using Equation (1) is defined by using the photocurrent produced by the AuNP-deposited chip, which is the ATP-sensing chip, as the base value. The recovery level is used as a quantitative analysis parameter for ATP detection.

**Figure 3:**
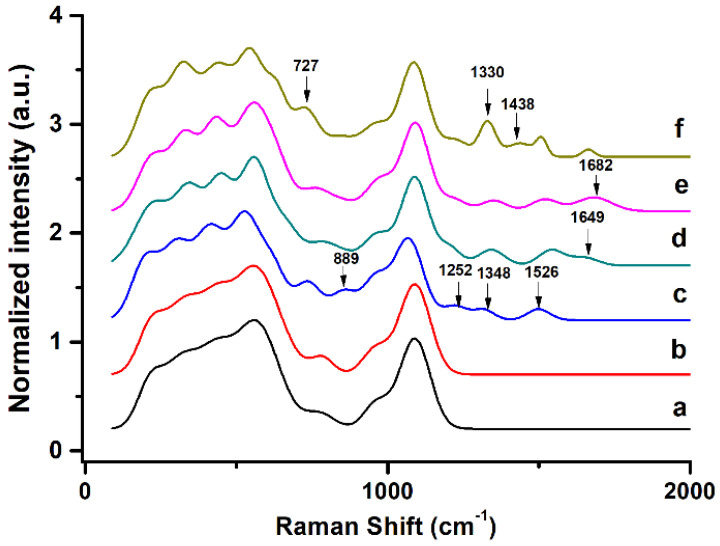
Raman spectra for ITO electrodes that are layer-by-layer fabricated with (**a**) APPA, (**b**) GO-OA complex linker, (**c**) b-PM, (**d**) ATP aptamer, (**e**) AuNP-labeled complementary strand and (**f**) ATP at the top.

Raman spectroscopy is used to determine the deposition of the fabricated material on each layer. Figure 3 shows the Raman spectra for ITO electrodes that are fabricated using different upper layers, with annotations showing the appearance of additional new peaks that are distinctly different from those for the previous layer. All spectra are deconvoluted using the PeakFit deconvolution program. The assignment of each band is listed in Table 1 based on previous studies.

As previously noted [11], the Raman spectrum for the electrode with an upper GO-OA linker layer is similar to that for the preceding one layered with APPA. The electrode that is coated with a b-PM monolayer features additional bands at 656, 889, 1252, 1348 and 1526 cm^−1^, which are respectively attributed to C-C twist, C-S stretches and Tyr of BR. Conjugation with the nucleic acid aptamer against ATP features a minor peak at 1649 cm^−1^. After hybridization with the AuNP-labeled complementary oligonucleotide, this peak is slightly red-shifted to 1682 cm^−1^, and its signal is augmented by the presence of AuNPs. Finally, the binding of ATP through the strand replacement mechanism is confirmed by the additional ATP characteristic bands at 727, 1330 and 1438 cm^−1^.

### 3.2. ATP Analysis Using Photocurrent Measurements

**Figure 4:**
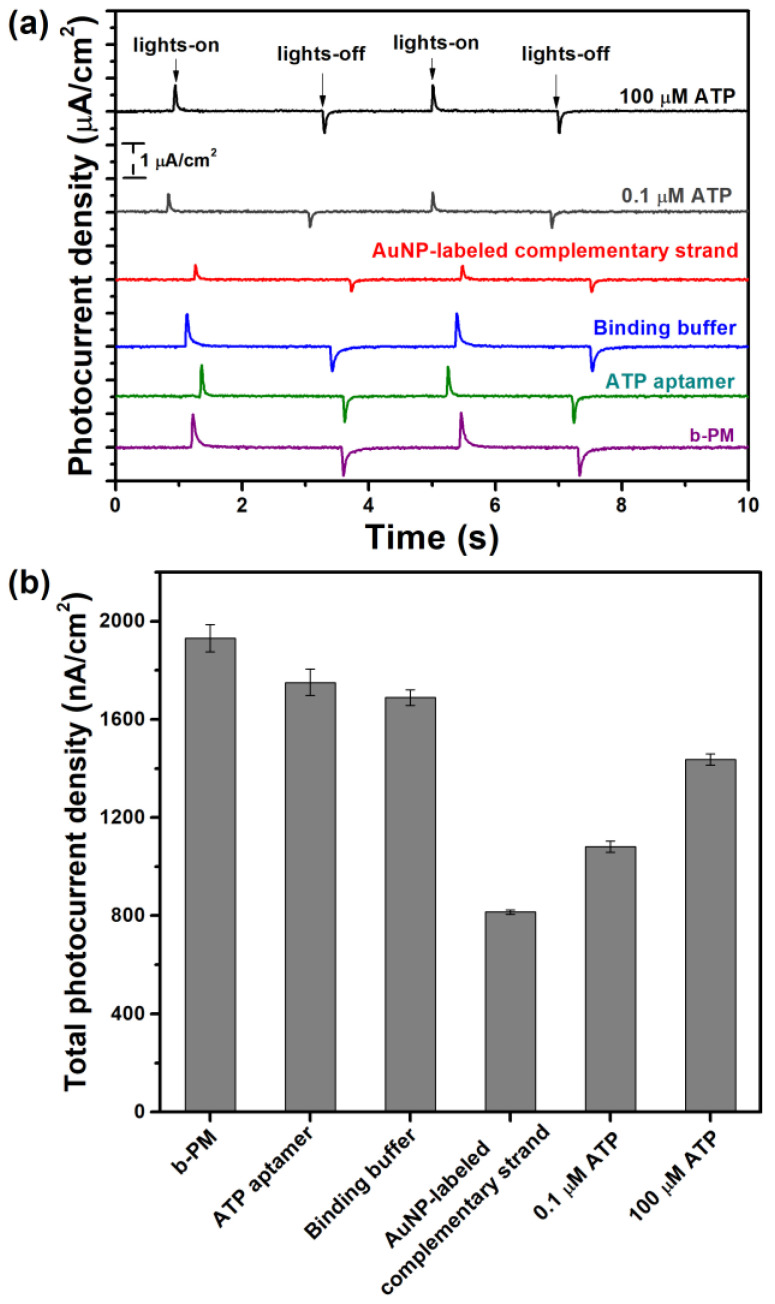
(**a**) Typical photocurrent responses and (**b**) total photocurrent density for chips that are fabricated using different upper layers. Lights-on and lights-off (**a**) show the response of a chip when irradiation is initiated and disrupted, respectively. All data are presented as the average value for three chips for a single type with one standard deviation (relative standard deviation, RSD < 3%).

The proposed method for ATP detection using BR as the photoelectric transducer is validated by measuring the photocurrent in the various top-layered b-PM chips. As shown in Figure 4a, all b-PM-coated chips generate similar photocurrent response profiles, which comprise two transient spikes with opposite polarities in response to lights-on and lights-off for the exciting laser. The peak intensity varies significantly across the response profiles.

Figure 4b compares the total photocurrent density for different chips. The pristine b-PM-coated electrode generates the greatest total photocurrent density, which decreases after the conjugation of the ATP aptamer on top, possibly because proton transportation is inhibited by the material that binds to the BR. The ensuing brief rinse with the ATP-binding buffer causes a slight decay in the total density value. However, when the ATP-aptamer-coated chip is hybridized with the AuNP-labeled complementary oligonucleotides, there is a significant decrease in the total photocurrent density. Therefore, hybridization is demonstrated to be successful, and the capability of the labeling AuNPs to partially hinder incident laser light is confirmed.

The peak intensity of the photocurrent response profile is partially restored when the resulting ATP-sensing chip is incubated with ATP solutions. The degree of restoration increases as the ATP concentration increases. The ATP-sensing chip exhibits the lowest total photocurrent density ($I_{AuNPs}$), which is less than the value for the rinsed ATP-aptamer-coated chip ($I_{buffer}$), after binding with AuNP-labeled complementary oligonucleotides. The difference between $I_{buffer}$ and $I_{AuNPs}$ is 100% for the photocurrent recovery calculation for the ATP-sensing chip to detect ATP, as shown in Equation (1).

**Figure 5:**
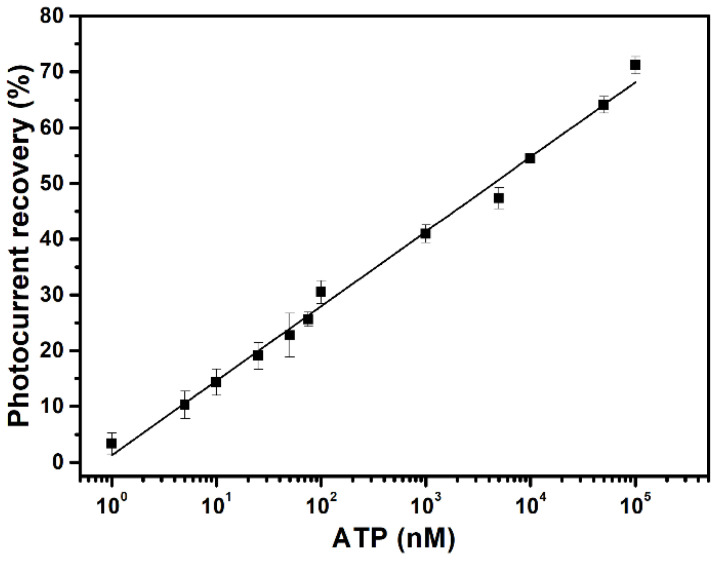
Calibration curve for ATP detection using ATP-sensing chip. All data are presented as the average value for three chips of a single type with one standard deviation (RSD < 4%).

Pure ATP solutions were quantitatively assayed with the as-prepared ATP-sensing chip, using concentrations of 0.1 nM to 0.1 mM. The 0.1 nM ATP solution results in a 0.9% recovery of the photocurrent, and the 1 nM ATP solution produces a 3.4% recovery. Increasing the ATP concentration gradually increases the degree to which the photocurrent recovers, as shown in Figure 5. There is a 71% recovery for 0.1 mM ATP. Solutions with a concentration of more than 0.1 mM may produce greater photocurrent recovery but are not used for this study because dilution is commonly practiced for sample preparation. The calibration curve (Figure 5) shows that the as-prepared ATP-sensing chip features good detection sensitivity and a wide dynamic detection range, so BR is suited for use as a signal transducer for single-step and sensitive ATP quantification because no further signal amplification is required.

### 3.3. Selectivity and Stability during Storage

To determine the selectivity of the as-prepared ATP-sensing chip, adenosine diphosphate (ADP) and other nucleoside triphosphates (NTPs) were tested in concentrations from 5 nM to 0.1 mM. ADP contains one less phosphate group that is bound to ribose than ATP. Cytidine triphosphate (CTP), guanosine triphosphate (GTP), thymidine phosphate (TTP) and uridine triphosphate (UTP) are NTPs with different nitrogenous base groups to ATP.

**Figure 6:**
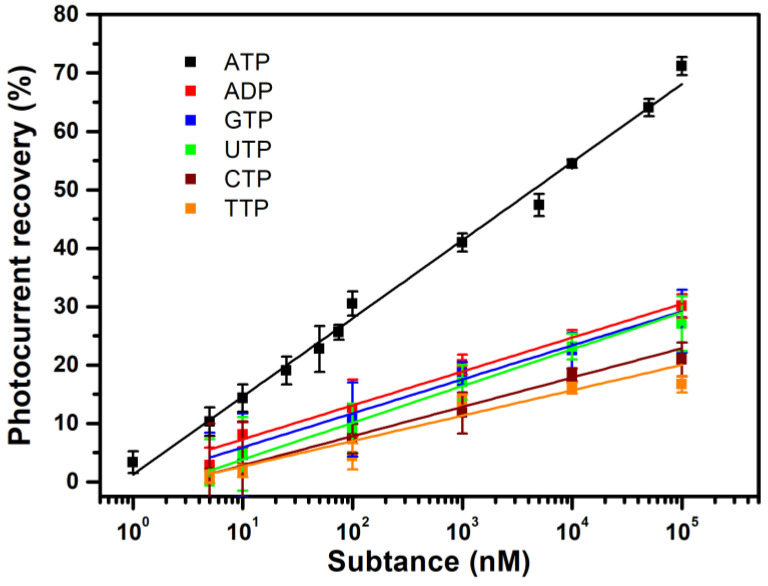
Calibration curve for the detection of ATP, ADP and other NTPs using the ATP-sensing chip: ATP (black), ADP (red), GTP (blue), UTP (green), CTP (wine) and TTP (orange). All data are presented as the average value for three chips of a single type with one standard deviation. RSD: ATP < 4%; ADP < 5%; GTP < 8%; UTP < 8%; CTP < 9%; TTP < 3%.

As shown in Figure 6, the ATP aptamer that is conjugated on the ATP-sensing chip also binds with these analogs, but it recognizes ATP much more efficiently. At 5 nM, the photocurrent recovery toward ATP is 10.3%, and the respective recovery toward ADP, CTP, GTP, TTP and UTP is only 1.3%, 1.0%, 1.0%, 1.0% and 0.0%. Photocurrent recovery also increases with concentration in the following order: ADP ≥ GTP ≈ UTP >> CTP > TTP. ADP and ATP contain the same adenine nitrogenous base, so ADP contributes to nonspecific binding. Similar to adenine, guanine is a purine base, so it is also probable that there is minor binding of GTP by the sensing chip. CTP, TTP and UTP all have pyrimidine nitrogenous base groups, so they feature the least nonspecific binding. The reason for the slightly higher value for UTP than that for CTP and TTP for nonspecific binding is unclear.

At a concentration of 0.1 mM, the photocurrent recovery toward ATP is 71.2%, but the respective recovery levels toward ADP, CTP, GTP, TTP and UTP are just 30.1%, 21.0%, 27.5%, 16.7% and 27.1%, which correspond to ATP concentrations of 144.1, 29.7, 91.6, 14.3 and 85.2 nM, according to the ATP calibration curve in Figure 5. These concentration values are much smaller than 0.1 mM, showing that interference in detection due to the presence of these analogs is insignificant for the proposed ATP-sensing chip. Selectivity is also high for other types of ATP aptasensors, including the AuNP-based colorimetric method, the electrochemical method and surface-enhanced Raman spectroscopy, which use the same ATP aptamer sequence as that used in this study [25,27,32]. The good selectivity for the proposed ATP aptasensor and those for previous studies shows that there is specific recognition between ATP and the used ATP aptamer.

**Figure 7:**
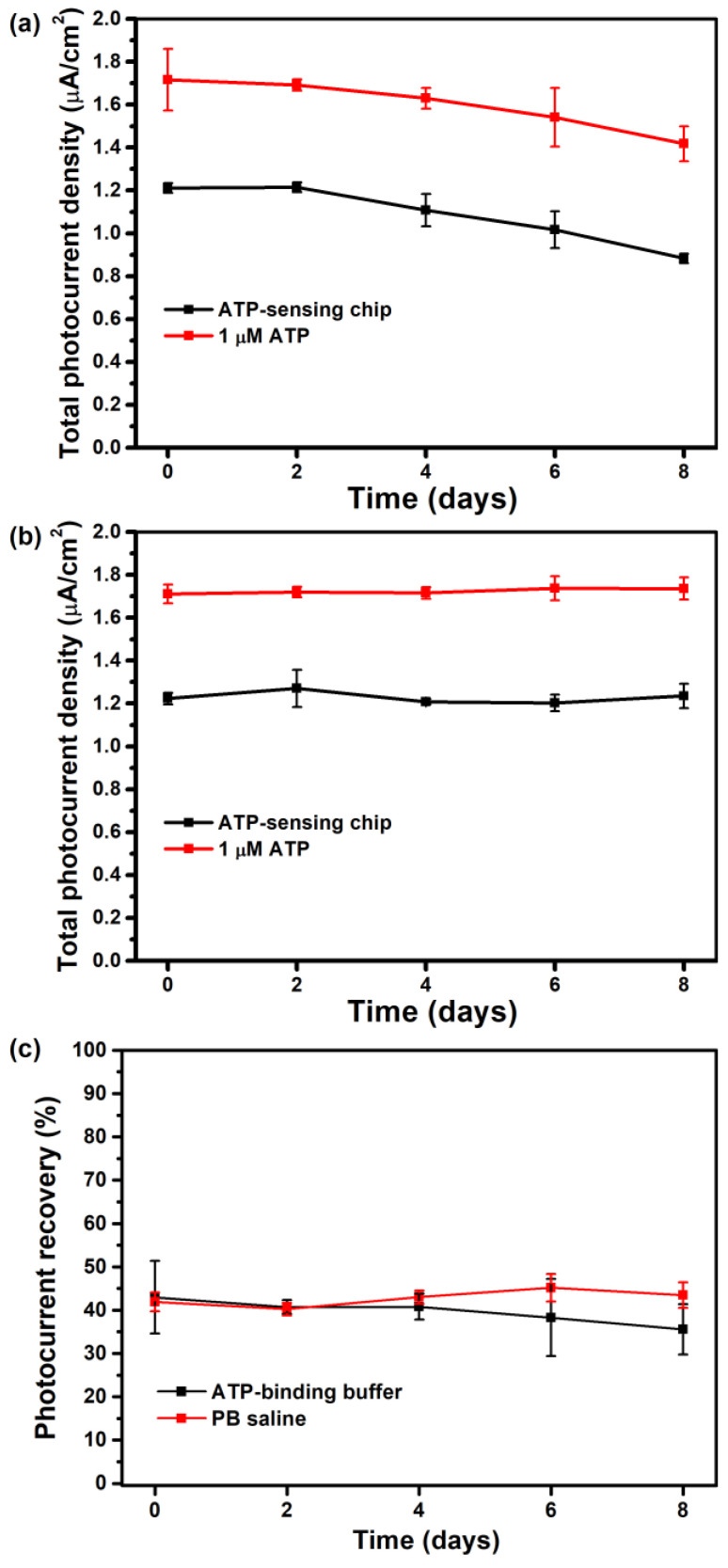
(**a**,**b**) Effect of storage time on the total photocurrent density for the ATP-sensing chip before (black) and after (red) incubation with 1 μM ATP using (**a**) the ATP-binding buffer (RSD < 9%) and (**b**) PB saline (RSD < 7%) as the storage buffer. (**c**) Effect of storage time on the photocurrent recovery for the ATP-sensing chip after binding with 1 μM ATP using the ATP-binding buffer (black) and PB saline (red) as the storage buffer. All data are presented as the average value for three chips of a single type with one standard deviation.

The stability of the ATP-sensing chip in storage was measured every other day. The chip was stored at 4 °C, either in an ATP-binding buffer containing 25 mM Tris-HCl and 300 mM NaCl at a pH of 8.2 or in a 10 mM phosphate buffer containing 150 mM NaCl at a pH of 8.0 (PB saline). As shown in Figure 7a, if the sensing chip is stored in the ATP-binding buffer, the photoelectric activity begins to decay on the fourth day (black). Surface-conjugated ATP aptamers remain active toward binding with 1 μM ATP, so the photocurrent is restored (red). However, the slight decrease and increased deviation in the photocurrent recovery (Figure 7c, black) show that the sensing chip gradually loses its ability to detect ATP, and its stability decreases when it is stored in the ATP-binding buffer. If the sensing chip is stored in the PB saline, the photoelectric activity and the ability to detect ATP are maintained for at least 8 days, and there is no significant reduction in photocurrent density or photocurrent recovery (Figure 7b,c, red).

### 3.4. Application for the Detection of Microorganisms

**Figure 8:**
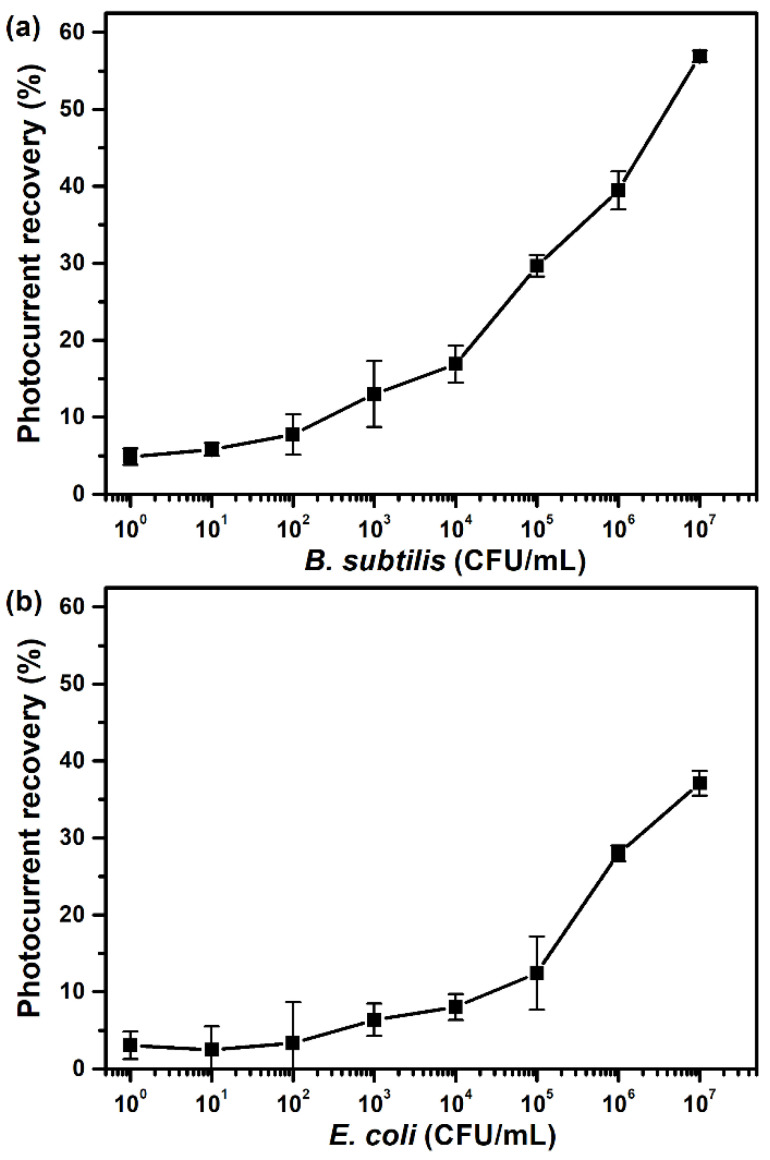
Relationship between the recovery of the photocurrent for an ATP-sensing chip on the cell concentration for (**a**) *B. subtilis* and (**b**) *E. coli* cultures. All data are presented as the average value for three chips of a single type with one standard deviation.

The as-prepared ATP-sensing chip was used to assay bacterial cultures that had been heat-lyzed and diluted at different concentrations. As shown in Figure 8, toward both the *B. subtilis* and *E. coli* cultures, the sensing chip restores the photocurrent as cell concentrations increase, so it detects variations in the amount of intracellular ATP in different test samples. This shows that the proposed ATP-sensing technology can be used to quantify a microorganism population.

The calibration curves for the *B. subtilis* and *E. coli* cultures in Figure 8 respectively resemble those for the *B. cereus* and *E. coli* cultures in a previous study that used bioluminescence ATP detection and signal enhancement via heat treatment [45]. The detection limit for *B. subtilis* for this study and the limit for *B. cereus* for a previous study is 10^2^ CFU/mL, and the detection limit for *E. coli* for both studies is 10^3^ CFU/mL. The results from both studies show that the signal intensity increases significantly if the concentration exceeds 10^4^ CFU/mL and 10^5^ CFU/mL for *Bacillus* species and *E. coli*, respectively. The detection sensitivity is greater for *Bacillus* species than for *E. coli* in both studies.

Previous studies have shown that *Bacillus* species and *E. coli* have a similar ATP content at a physiological pH [46,47], but one study showed that the efficacy of ATP-based microbial detection methods is limited for Gram-negative bacteria because cell lysis is incomplete [48]. The detection sensitivity for the proposed system will be increased in the future using better cell lysis methods, such as the addition of lysozymes or the use of more efficient lysis buffers.

### 3.5. Application for the Detection of Spiked Field Samples

**Figure 9:**
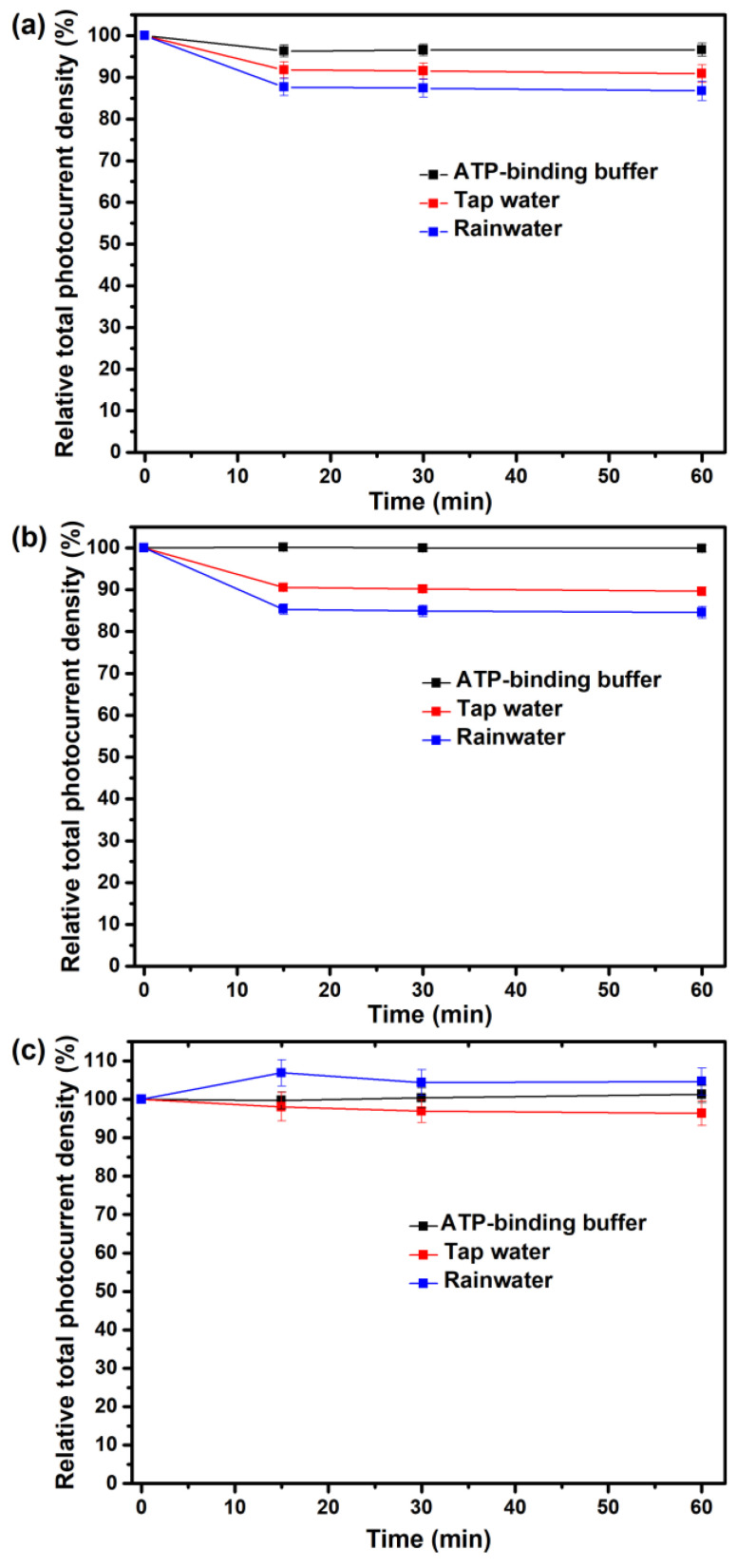
Effect of incubation time on the relative total photocurrent density for (**a**) the pristine b-PM chip, (**b**) the ATP-aptamer-coated chip and (**c**) the ATP-sensing chip in (black) the ATP-binding buffer, (red) tap water and (blue) ground-harvested rainwater. The values of the total photocurrent densities generated by each kind of chip before incubation were taken as 100%, respectively, in each figure. All data are presented as the average value for three chips of a single type with one standard deviation.

To investigate the feasibility of the ATP-sensing chip in practical applications, the amount of ATP in the spiked field samples of tap water and ground-harvested rainwater was measured and compared with that in the spiked ATP-binding buffer. As shown in Figure 9a,b, the photocurrent production of the pristine b-PM chip and the ATP-aptamer-coated chip both notably reduce to constant levels after incubation in tap water and rainwater for 15 min, possibly because they are more acidic and complex than the ATP-binding buffer. The pH values of the ATP-binding buffer, tap water and rainwater are 8.20, 7.16, and 5.27, respectively. In addition, the composition of ground-harvested rainwater from the densely populated and heavy-traffic Taipei City is obviously much more complex than that of tap water. Therefore, the effect of rainwater on the photoelectric activities of the chips is more significant.

**Figure 10:**
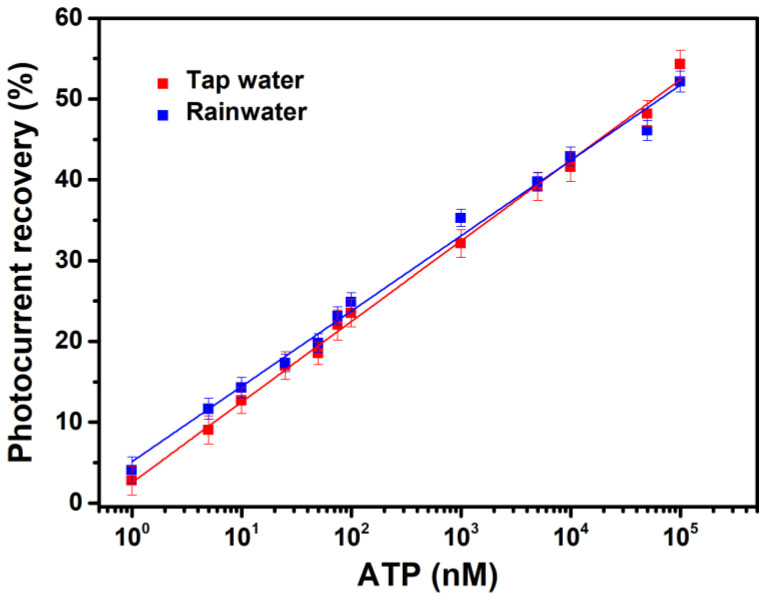
Photocurrent recovery for the ATP-sensing chip as a function of ATP concentrations in (red) tap water and (blue) ground-harvested rainwater. All data are presented as the average value for three chips of a single type with one standard deviation. RSD: tap water < 4%; rainwater < 5%.

Nevertheless, the patterns of the calibration curves of tap water and rainwater toward ATP (Figure 10) are like that of the ATP-binding buffer (Figure 5), all exhibiting a linear relationship between photocurrent recovery levels and ATP concentrations on a linear–log plot. The linear regression R^2^ values of the ATP-binding buffer, tap water and rainwater are 0.998, 0.996, and 0.991, respectively. The y-intercept of the regression line of rainwater is slightly higher than that of tap water, implying contamination by microbes in the harvested rainwater. This slight background ATP amount also contributes to the higher photocurrent values (>100%) in the rainwater data of Figure 9c.

Using those calibration curves, the spiked amounts of ATP in the standard ATP-binding buffer and those two field samples were determined. The results are shown in Table 2. The RSD values at 30 nM and 300 nM ATP are very low for all three cases, suggesting the high reproducibility of the ATP-sensing chip in field sample tests. The recovery ratios are between 96% and 104% with the ATP-binding buffer, between 93% and 115% with tap water and between 93% and 120% with rainwater. The recovery ratios increase with the added ATP amounts in the latter two cases. The decrease in measurement accuracy with increasing ATP concentrations may be attributed to the semi-log linear relationship of the calibration curve using the current development. In addition, because the recovery ratios with the ATP-binding buffer are all close to 100%, the effect of these two field samples on BR photoelectric activity could have also caused lower measurement accuracy. To yield a recovery ratio close to 100%, as obtained in the previous ATP detection study using a personal glucose meter [49], another ATP-sensing chip with a linear calibration curve and improved photoelectric stability will be beneficial in the future.

## 4. Conclusions

A selective, sensitive, single-step and simple ATP detection system is demonstrated, which allows the direct quantification of the amount of ATP in 30 min, without the need for sample pretreatment or post-detection signal amplification. The proposed reverse detection method uses AuNP labeling and specific DNA strand displacement to enhance the signal, so the b-PM monolayer photoelectric transducer responds to the presence of ATP by restoring its photocurrent. Photocurrent recovery increases as the ATP concentration increases. This detection principle and technique can be used for other small molecules, for which binding nucleic acid aptamers are available.

In conjunction with previous methods for the detection of Hb, HbA1c and microorganisms, which also use a b-PM monolayer as the photoelectric transducer, color proteins, microbes and small molecules in samples for environmental monitoring, food contamination risk assessment, health diagnosis and other applications can also be assayed using this BR-based sensing platform.

## Figures and Tables

**Table 1:** Raman band (cm^−1^) assignment for ITO electrodes that are fabricated using different upper layers ^a^.

APPA	GO-OA	b-PM	ATP Aptamer	AuNP-Labeled Complementary Strand	ATP	Band Assignment	References
212	215	235	226	222	216		
321	333	337	346	330	320		
433	437	447	448	430	438		
565	560	564	557	554	551		
		656	658	643	633	C-C twist/C-S stretches/Tyr	[35,36,37]
777	785	769	775	758			
					727	ATP (ring-breathing of adenine ring)	[38]
			841	845	845	DNA bases	[36]
		889				Tyrosine doublet	[35,37,39,40]
963	962	990	966	975	963		
1090	1090	1099	1090	1093	1088		
		1252	1204	1218	1224	DNA bases/retinal	[36,41,42]
		1348	1343	1351	1330	ATP (C5-N7 stretching)/DNA bases/retinal	[36,38,42,43,44]
					1438	ATP (C=N stretching)	[38]
		1526	1541	1519	1510	retinal	[42]
			1649	1682	1664	DNA bases	[36]

^a^ Data originate from the deconvoluted Raman spectra, which are derived from Figure 3.

**Table 2:** Determination of the ATP amount spiked in the ATP-binding buffer, tap water and ground-harvested rainwater.

Added ATP (nM)	Measured ATP ^a^ (nM)	SD (nM)	RSD (%)	Recovery ^b^ (%)
ATP-binding buffer				
3	3.12	0.068	2.18	104.0
30	31.05	0.096	0.31	103.5
300	289.57	0.075	0.03	96.5
Tap water				
3	2.89	0.17	5.97	96.3
30	32.20	0.16	0.51	107.3
300	343.8	0.14	0.04	114.6
Ground-harvested rainwater				
3	2.81	0.17	6.01	93.6
30	32.6	0.14	0.44	108.7
300	360.25	0.13	0.04	120.1

^a^ Average value for three chips for a single condition. ^b^ Measured value/added value × 100.

## Data Availability

No new data were created or analyzed in this study. Data sharing is not applicable to this article.
